# Comparing head muscles among Drusinae clades (Insecta: Trichoptera) reveals high congruence despite strong contrasts in head shape

**DOI:** 10.1038/s41598-022-04790-2

**Published:** 2022-01-20

**Authors:** Carina Zittra, Simon Vitecek, Thomas Schwaha, Stephan Handschuh, Jan Martini, Ariane Vieira, Hendrik C. Kuhlmann, Johann Waringer

**Affiliations:** 1 Unit Limnology, Department of Functional and Evolutionary Ecology, University of Vienna, Djerassiplatz 1, 1030 Vienna, Austria; 2 WasserCluster Lunz - Biologische Station GmbH, Dr. Carl Kupelwieser Promenade 5, 3293 Lunz am See, Austria; 3 Institute of Hydrobiology and Aquatic Ecosystem Management, University of Natural Resources and Life Sciences, Vienna Gregor-Mendel-Straße 33, 1180 Vienna, Austria; 4 Department of Evolutionary Biology, University of Vienna, Djerassiplatz 1, 1030 Vienna, Austria; 5 VetCore Facility for Research Imaging Unit, Vetmeduni Vienna, Veterinaerplatz 1, 1210 Vienna, Austria; 6 Department of Ecology, University of Innsbruck, Technikerstraße 25, 6020 Innsbruck, Austria; 7 Institute of Fluid Mechanics and Heat Transfer, TU Wien, Getreidemarkt 9, 1060 Vienna, Austria

**Keywords:** Entomology, Limnology

## Abstract

The subfamily Drusinae (Limnephilidae, Trichoptera) comprises a range of species exhibiting differently shaped head capsules in their larval stages. These correspond to evolutionary lineages pursuing different larval feeding ecologies, each of which uses a different hydraulic niche: scraping grazers and omnivorous shredders sharing rounded head capsules and filtering carnivores with indented and corrugated head capsules. In this study, we assess whether changes in head capsule morphology are reflected by changes in internal anatomy of Drusinae heads. To this end, internal and external head morphology was visualized using µCT methods and histological sections in three Drusinae species—*Drusus franzi*, *D*. *discolor* and *D*. *bosnicus—*representing the three evolutionary lineages. Our results indicate that Drusinae head musculature is highly conserved across the evolutionary lineages with only minute changes between taxa. Conversely, the tentorium is reduced in *D*. *discolor*, the species with the most aberrant head capsule investigated here. Integrating previous research on Drusinae head anatomy, we propose a fundamental Drusinae blueprint comprising 29 cephalic muscles and discuss significance of larval head capsule corrugation in Trichoptera.

## Introduction

Nature comes in manifold color, smell, shape, and taste. As in all other life forms a great variety of different morphologies can be observed in aquatic insects as well^1^. Differences in morphology typically are accompanied by a distinct ecological niche, related to—for instance—feeding mode^2^. Predators among aquatic insects may enjoy prehensile mouthparts, or a slender agile body that allows them to pounce on their prey^3^. Grazers have developed their mouthparts to an intricate array of brushes and bristles to scrape benthic algae^3^. Passive filter-feeders, on the other hand, often have elongated antennae or legs equipped with bristles to collect food particles from the flow^3^. And those among aquatic insects feeding on larger detritus, the so-called shredders, often are rather unremarkable but equipped with robust mandibles to masticate their food^3^.

Naturally, species of these functional feeding groups occur in different patches in a local habitat—following the distribution of food sources in the stream bed^2^. Predators can pick any spot if prey densities are high enough, but grazers, filter-feeders and shredders need to select specific habitat patches to maximize feeding efficiency^4–7^. These microhabitats are most importantly defined by distinct flow velocities: at slowly flowing sections detritus accumulates whereas benthic algae grow most densely on the upper sides of large stones in faster waters, where also food particle density in the drift is high^8–12^.

Consequently, adaptations to hydraulic stress can be observed in aquatic insects in addition to those enforced by feeding modes. In caddisflies, behavioural adaptations include the use of silk as safety tether in Rhyacophilidae, Hydropsychidae and Brachycentridae, ballast stones in Goeridae^4^, and a silken stalk in Limnocentropodidae^3^. Curiously, morphological adaptations to hydraulic stress are rarely considered in caddisflies. The bodies of other aquatic insects are often modified to reduce of hydraulic stress. This is demonstrated by the flattened, streamlined bodies of Heptageniidae or Psephenidae, that certainly are at the pinnacle of morphological adaptation to hydraulic stress^3^.

**Figure 1:**
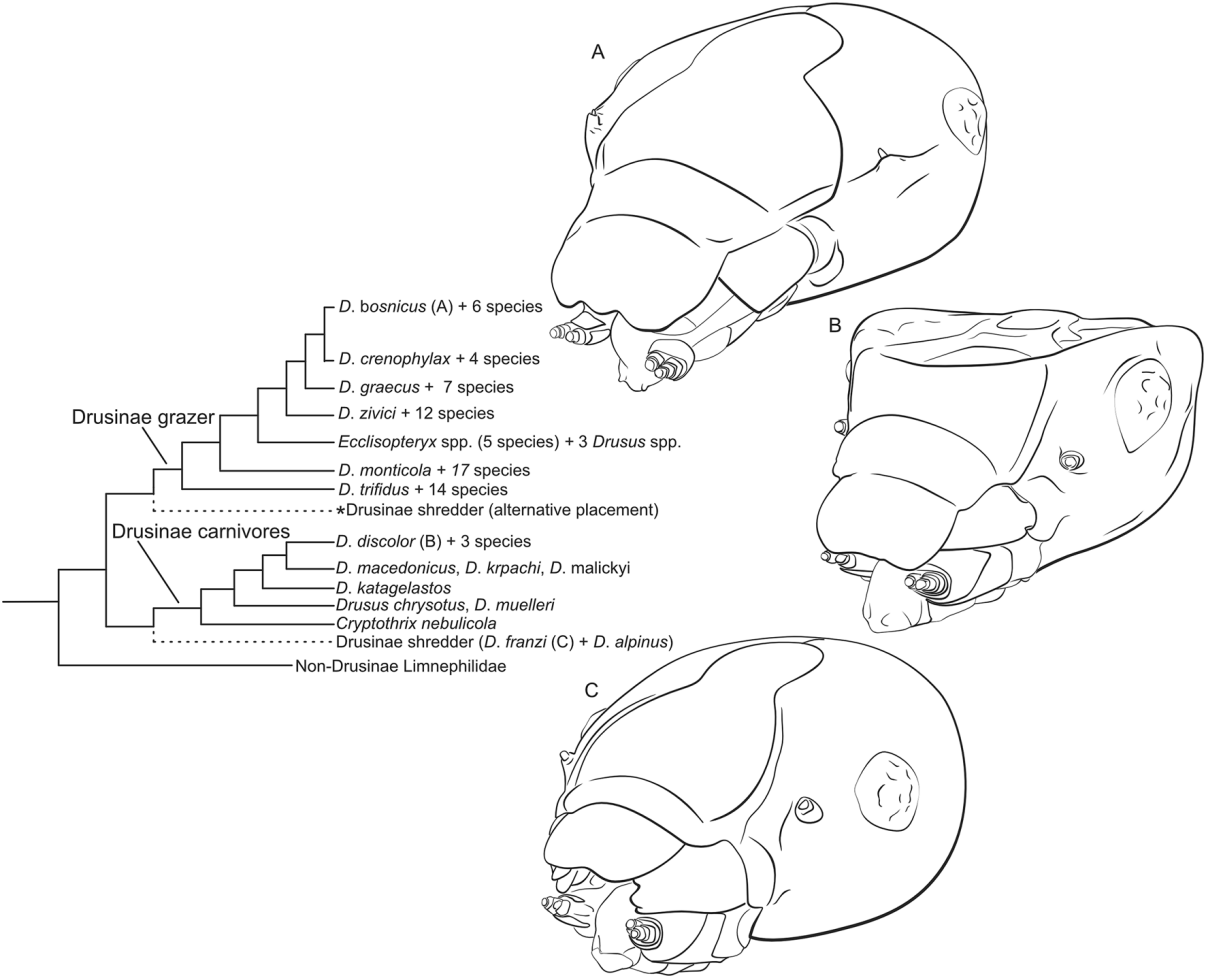
Relationships of Drusinae evolutionary lineages based on published phylogenies^13,14^. Drusinae scraping grazers and Drusinae filtering carnivores are monophyla; placement of Drusinae shredders is equivocal as sister of either Drusinae grazers or Drusinae carnivores. Gross head capsule morphology of Drusinae grazers (**A**, *Drusus bosnicus*) and Drusinae shredders (**C**, *D*. *franzi*) is quite similar, and differs distinctly from that of Drusinae carnivores (**B**, *D*. *discolor*). Figures not to scale; del. Vitecek. This figure was built using the Affinity Suite, version 1.10.4 (Serif Europe Ltd., https://affinity.serif.com/).

Recently, head capsule morphology was identified as potential adaptation to hydraulic stress and a particular feeding mode in Drusinae caddisflies^13^. The Drusinae are an intriguing group of Limnephilidae, and comprise three distinct evolutionary clades^13–15^. Each of these clades comprises species sharing a particular larval feeding ecology: scraping grazers with toothless mandibles, and filtering carnivores and shredders with tooth-bearing mandibles. While evolutionary relationships among these clades remain to be clarified, comparative morphological analyses based on adults and larvae indicate that Drusinae shredders may have retained ancestral characters^14^. Most probably, Drusinae shredders are sister to Drusinae carnivores, with Drusinae grazers as sister to a combined Drusinae carnivore and shredder clade (Fig. 1)^13^. Drusinae likely diversified in vicariant conditions under the impact of repeated ice ages and geological processes with scraping grazers representing the greatest radiation of the group^13,16,17^. Extant Drusinae bear marks of their evolutionary background and develop adult and larval characters that indicate to which feeding group each species belongs^13^. In Drusinae larvae, head capsule morphology is of particular importance: head capsules of species of the filtering carnivore clade differ strikingly from the rounded head capsules of their congeners and other European Limnephilidae^13^. The significance of head capsule shape for their ecology remains unclear, but a link to feeding ecology was recently proposed. And while the cephalic anatomy of *Drusus trifidus* (Mclachlan 1868) and *D*. *monticola* Mclachlan 1876 is known^18,19^, there is to date no information whether these scraping grazer species were an evolutionary stemgroup or representative for all other Drusinae, including shredders and the filtering carnivores with their peculiar heads (Fig. 1).

We propose that the aberrant forms of filtering carnivore Drusinae heads are reflected in modifications of the internal anatomy, which, in turn, correspond to different evolutionary trends within Drusinae. To address our hypotheses, we used a sample of three different species comprising a shredder (*D*. *franzi* Schmid 1956), a filtering carnivore (*D*. *discolor* (Rambur 1842)) and a scraping grazer (*D*. *bosnicus* Klapálek 1900), and integrated available information on *D*. *trifidus* and *D*. *monticola*. We hypothesized that location and number (or volume) of head muscles differs among shredder, scraping grazer, and filtering carnivore Drusinae. In particular, we posit that the shredder species will have the most complex internal organization, and that shifts in attachment sites as well as numbers of muscles occur in grazers and filtering carnivores.

## Results

**Figure 2:**
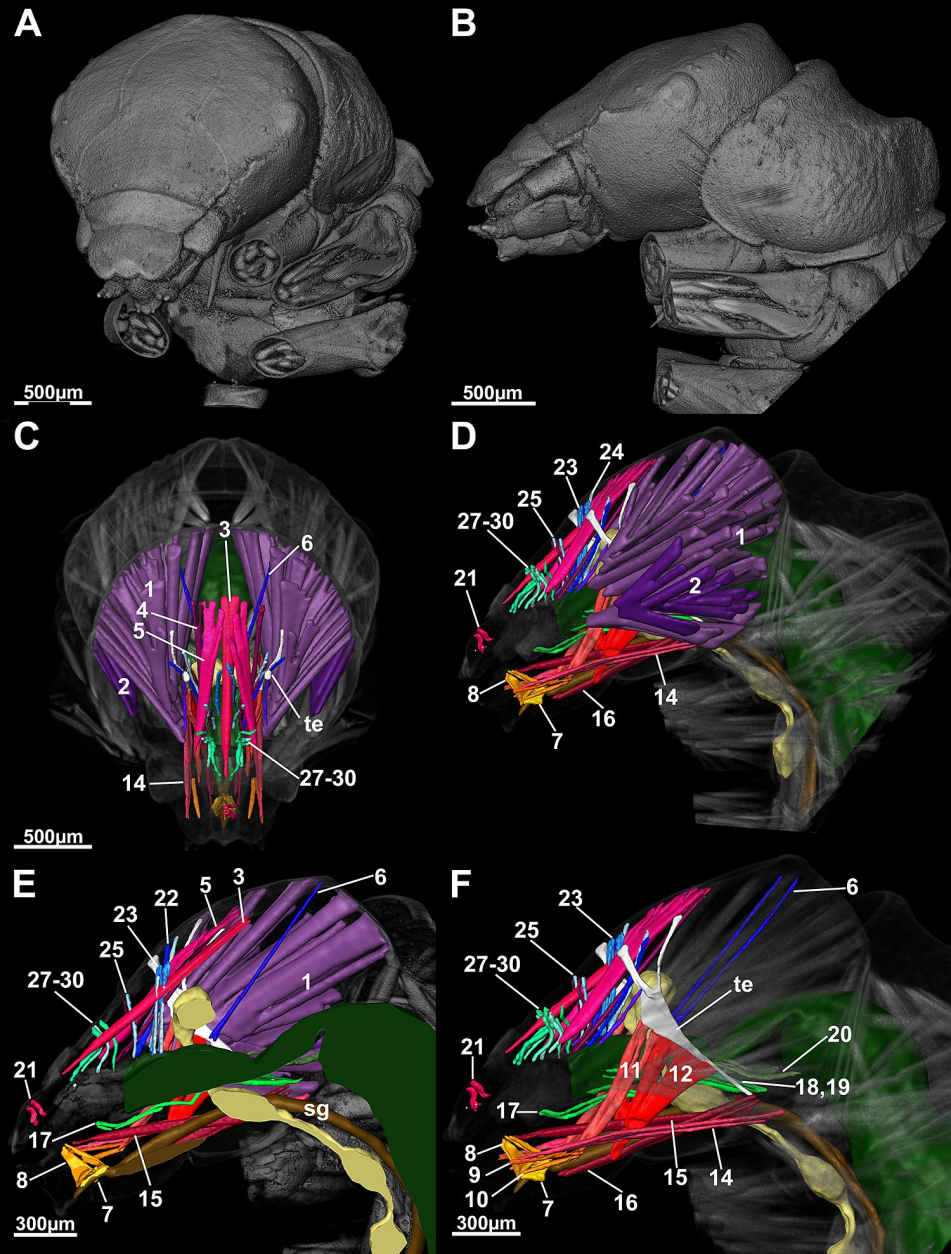
3D-reconstruction of the morphology (**A**, left fronto-lateral view, **B**, lateral view) and the internal head anatomy (**C**, frontal view, **D**, lateral view, **E**, sagittal view, **F**, parasagittal plane at the level of the tentorium) of *Drusus bosnicus* (5th larval instar) based on µCT data. Abbreviations: 1 = Musculus (M.) cranio-mandibularis medialis, 2 = M. cranio-mandibularis lateralis, 3 = M. fronto-labralis, 4 = M. fronto-pharyngalis, 5 = M. fronto-epipharyngalis, 6 = M. cranio-postpharyngalis dorsalis, 7 = M. praemento-salivaris, 8 = M. hypopharyngo-salivarialis, 9. = M. basistipito-dististipitalis lateralis, 10 = M. basistipito-dististipitalis medialis, 11 = M. tentorio-stipitalis, 12 = M. tentorio-cardinalis, 14 = M. cranio-dististipitalis, 15 = M. cranio-praementalis medialis, 16 = M. cranio-praementalis lateralis, 17 = M. cranio-cibarialis, 18 = M. cranio-pharyngalis anterior, 19 = M. cranio-pharyngalis posterior, 20 = M. cranio-postpharyngalis ventralis, 21 = M. labro-epipharyngalis, 22 = M. cranio-pharyngalis lateralis, 23 = M. fronto-pharyngalis medialis, 24 = M. fronto-pharyngalis lateralis, 25 and 26 = M. fronto-pharyngalis ventralis, 27 and 28 = M. clypeo-pharyngalis, 29 and 30 = M. clypeo-cibarialis, te = tentorium. The figure was built using the visualization software Amira 2020.2 (Thermo Fisher Scientific, https://www.thermofisher.com).

**Figure 3:**
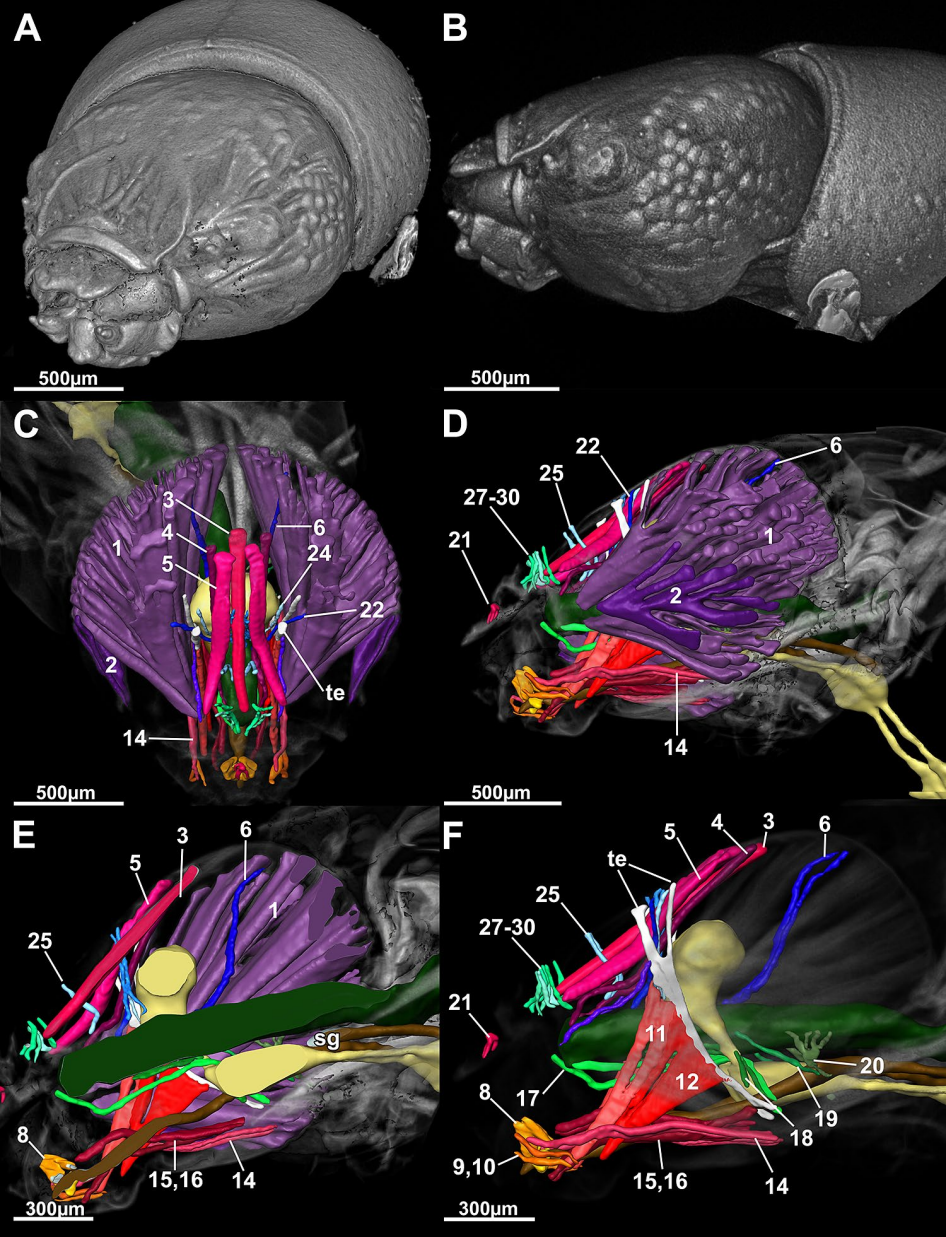
3D-reconstruction of the morphology (**A**, left fronto-lateral view, **B**, lateral view) and the internal head anatomy (**C**, frontal view, **D**, lateral view, **E**, sagittal view, **F**, parasagittal plane at the level of the tentorium) of *Drusus franzi* (5th larval instar) based on µCT data. Abbreviations: 1 = Musculus (M.) cranio-mandibularis medialis, 2 = M. cranio-mandibularis lateralis, 3 = M. fronto-labralis, 4 = M. fronto-pharyngalis, 5 = M. fronto-epipharyngalis, 6 = M. cranio-postpharyngalis dorsalis, 7 = M. praemento-salivaris, 8 = M. hypopharyngo-salivarialis, 9. = M. basistipito-dististipitalis lateralis, 10 = M. basistipito-dististipitalis medialis, 11 = M. tentorio-stipitalis, 12 = M. tentorio-cardinalis, 14 = M. cranio-dististipitalis, 15 = M. cranio-praementalis medialis, 16 = M. cranio-praementalis lateralis, 17 = M. cranio-cibarialis, 18 = M. cranio-pharyngalis anterior, 19 = M. cranio-pharyngalis posterior, 20 = M. cranio-postpharyngalis ventralis, 21 = M. labro-epipharyngalis, 22 = M. cranio-pharyngalis lateralis, 23 = M. fronto-pharyngalis medialis, 24 = M. fronto-pharyngalis lateralis, 25 and 26 = M. fronto-pharyngalis ventralis, 27 and 28 = M. clypeo-pharyngalis, 29 and 30 = M. clypeo-cibarialis, te = tentorium. The figure was built using the visualization software Amira 2020.2 (Thermo Fisher Scientific, https://www.thermofisher.com).

**Figure 4:**
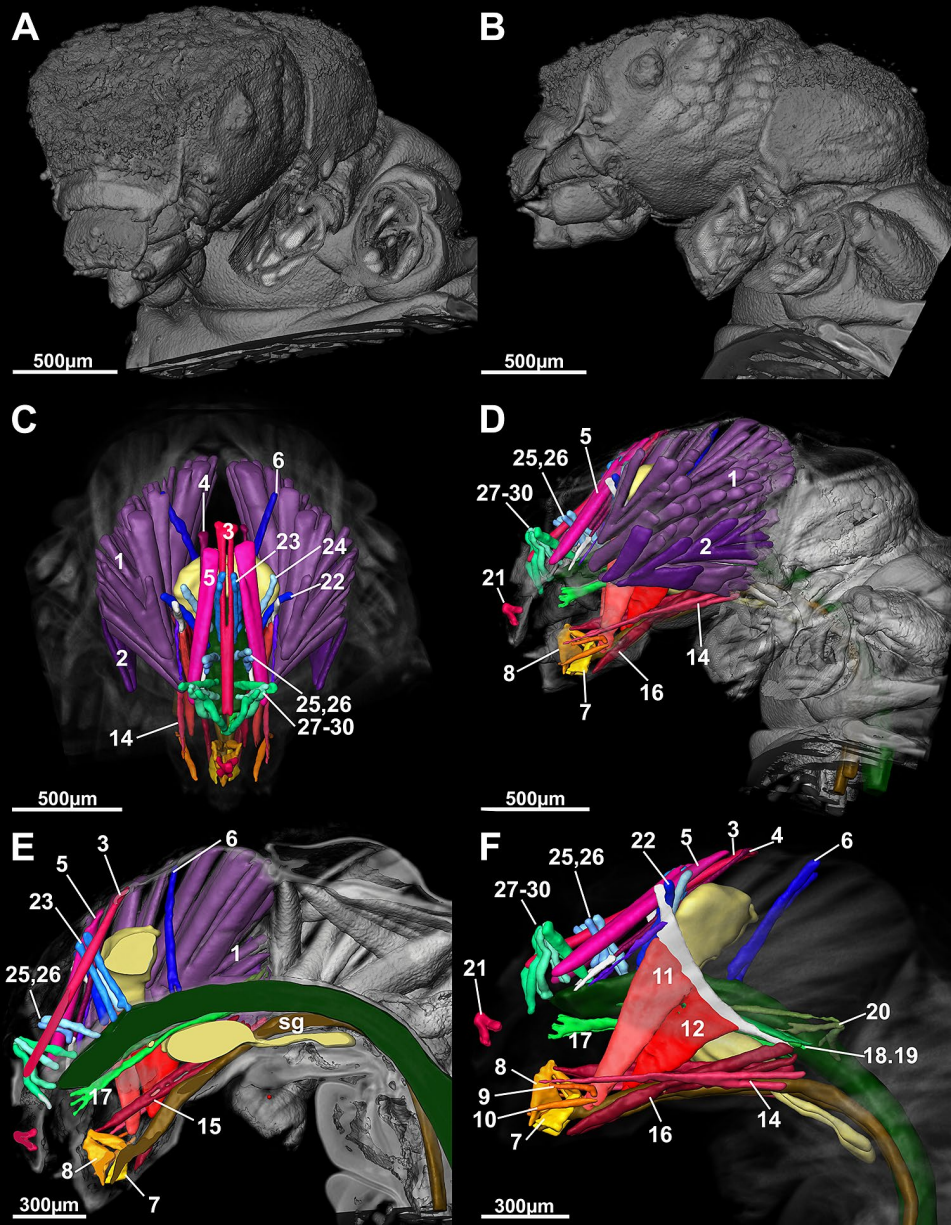
3D-reconstruction of the morphology (**A** left fronto-lateral view, **B** lateral view) and the internal head anatomy (**C** frontal view, **D** lateral view, **E** sagittal view, **F** Hagenparasagittal plane at the level of the tentorium) of *Drusus discolor* (5th larval instar) based on µCT data. Abbreviations: 1 = Musculus (M.) cranio-mandibularis medialis, 2 = M. cranio-mandibularis lateralis, 3 = M. fronto-labralis, 4 = M. fronto-pharyngalis, 5 = M. fronto-epipharyngalis, 6 = M. cranio-postpharyngalis dorsalis, 7 = M. praemento-salivaris, 8 = M. hypopharyngo-salivarialis, 9. = M. basistipito-dististipitalis lateralis, 10 = M. basistipito-dististipitalis medialis, 11 = M. tentorio-stipitalis, 12 = M. tentorio-cardinalis, 14 = M. cranio-dististipitalis, 15 = M. cranio-praementalis medialis, 16 = M. cranio-praementalis lateralis, 17 = M. cranio-cibarialis, 18 = M. cranio-pharyngalis anterior, 19 = M. cranio-pharyngalis posterior, 20 = M. cranio-postpharyngalis ventralis, 21 = M. labro-epipharyngalis, 22 = M. cranio-pharyngalis lateralis, 23 = M. fronto-pharyngalis medialis, 24 = M. fronto-pharyngalis lateralis, 25 and 26 = M. fronto-pharyngalis ventralis, 27 and 28 = M. clypeo-pharyngalis, 29 and 30 = M. clypeo-cibarialis, te = tentorium. The figure was built using the visualization software Amira 2020.2 (Thermo Fisher Scientific, https://www.thermofisher.com).

**Figure 5:**
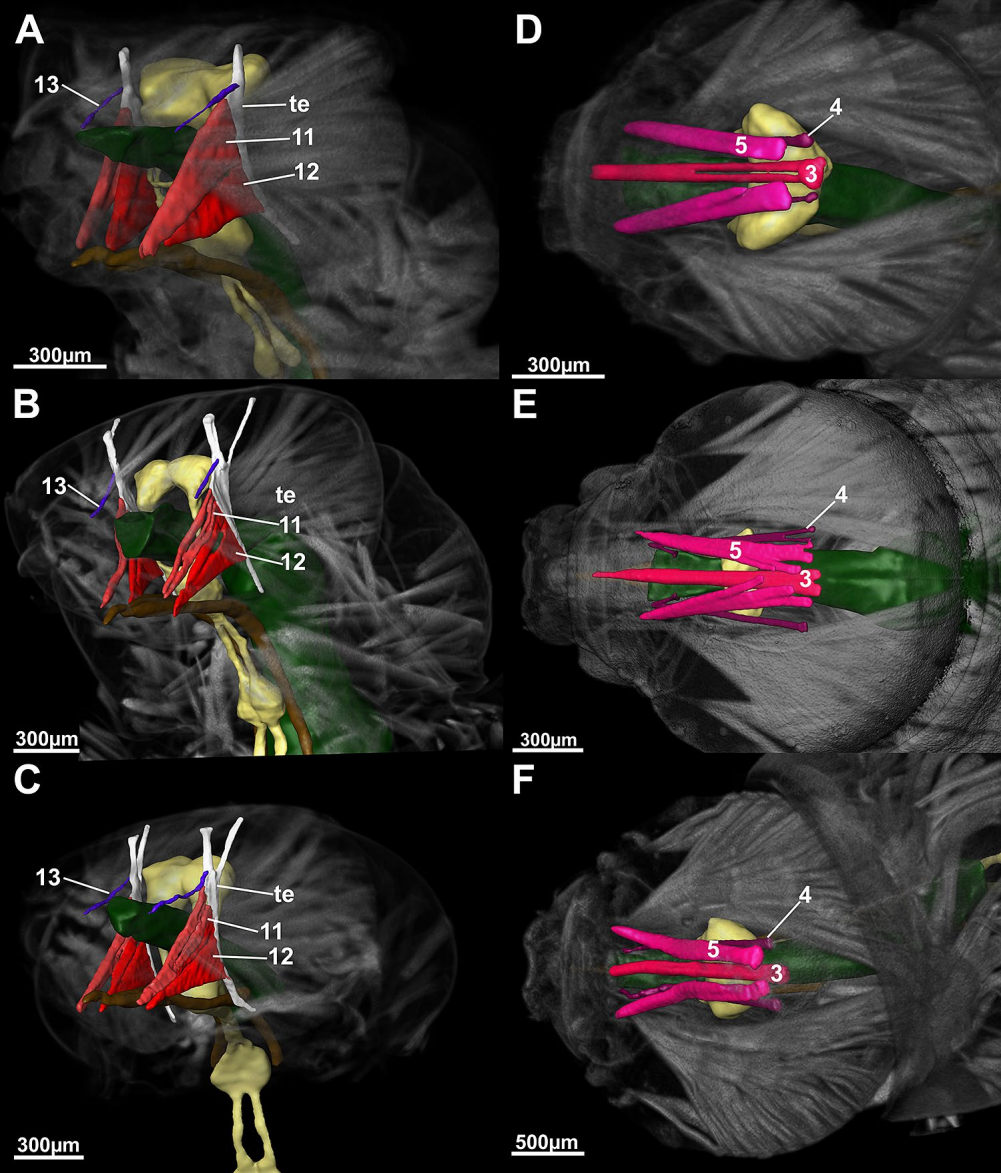
Comparison of the left ventro-lateral (**A**–**C**) and frontal view (**D**–**F**) of 5th instar larvae of *Drusus discolor* (**A**, **D**), *D. bosnicus* (**B**, **E**) and *D. franzi* (**C**, **F**) based on µCT data. Abbreviations: 3 = Musculus fronto-labralis, 4 = M. fronto-pharyngalis, 5 = M. fronto-epipharyngalis, 11 = M. tentorio-stipitalis, 12 = M. tentorio-cardinalis, 13 = M. tentorio-mandibularis, te = tentorium. The figure was built using the visualization software Amira 2020.2 (Thermo Fisher Scientific, https://www.thermofisher.com).

Our first aim was to define a generalized Drusinae head. We found, in brief, the general Drusinae head to comprise a tentorium with 2 branches and a set of 29 cephalic muscles to operate mouthparts and the alimentary canal (Figs. 2, 3, 4, 5). The largest muscles in the Drusinae head operate the mandibles: the Musculus cranio-mandibularis medialis (1, the adductor) and the M. cranio-mandibularis lateralis (2, the abductor); an additional mandibular muscle originates from the tentorium (M. tentorio-mandibularis; 13). From the frontoclypeus, three pairs of muscles originate that insert in the labrum (M. fronto-labralis; 3), the pharynx (M. fronto-pharyngalis; 4) and the epipharynx (M. fronto-epipharyngalis; 5). The maxillolabium has four pairs of intrinsic muscles: The M. praemento-salivaris (7), the M. hypopharyngo-salivarialis (8), the M. basistipito-dististipitalis lateralis (9), and the M. basistipito-dististipitalis medialis (10). Further, a set of muscles originates from the tentorium and comprises the M. tentorio-stipitalis (11) and the M. tentorio-cardinalis (12) that insert in the maxillolabium at stipes/cardo, and the previously mentioned M. tentorio-mandibularis (13) (Fig. 5). Close to the base of the tentorium, three muscles originate that also insert in the maxillolabium: The M. cranio-dististipitalis (14), the M. cranio-praementalis medialis (15) and the M. cranio-praementalis lateralis (16). Besides these muscle groups that grant movement to the mouthparts, the alimentary canal is operated and held in position by several thin muscle bundle pairs. On the ventral side of the pharynx is inserted the M. cranio-cibarialis (17), the M. cranio-pharyngalis anterior and posterior (18, 19), and the M. cranio-postpharyngalis ventralis (20). Dorsally, function and position of the alimentary canal is supported by the M. labro-epipharyngalis (21), the M. cranio-pharyngalis lateralis (22), the M. fronto-pharyngalis medialis (23), the M. fronto-pharyngalis lateralis (24), the M. fronto-pharyngalis ventralis (25 [or 25, 26; split into two muscle bundles in *D*. *discolor*]), the M. clypeo-pharyngalis (27, 28; split into two muscle bundles), and the M. clypeo-cibarialis (29, 30; split into two muscle bundles) in front of the brain, and the M. cranio-postpharyngalis dorsalis (6) behind the brain.

**Figure 6:**
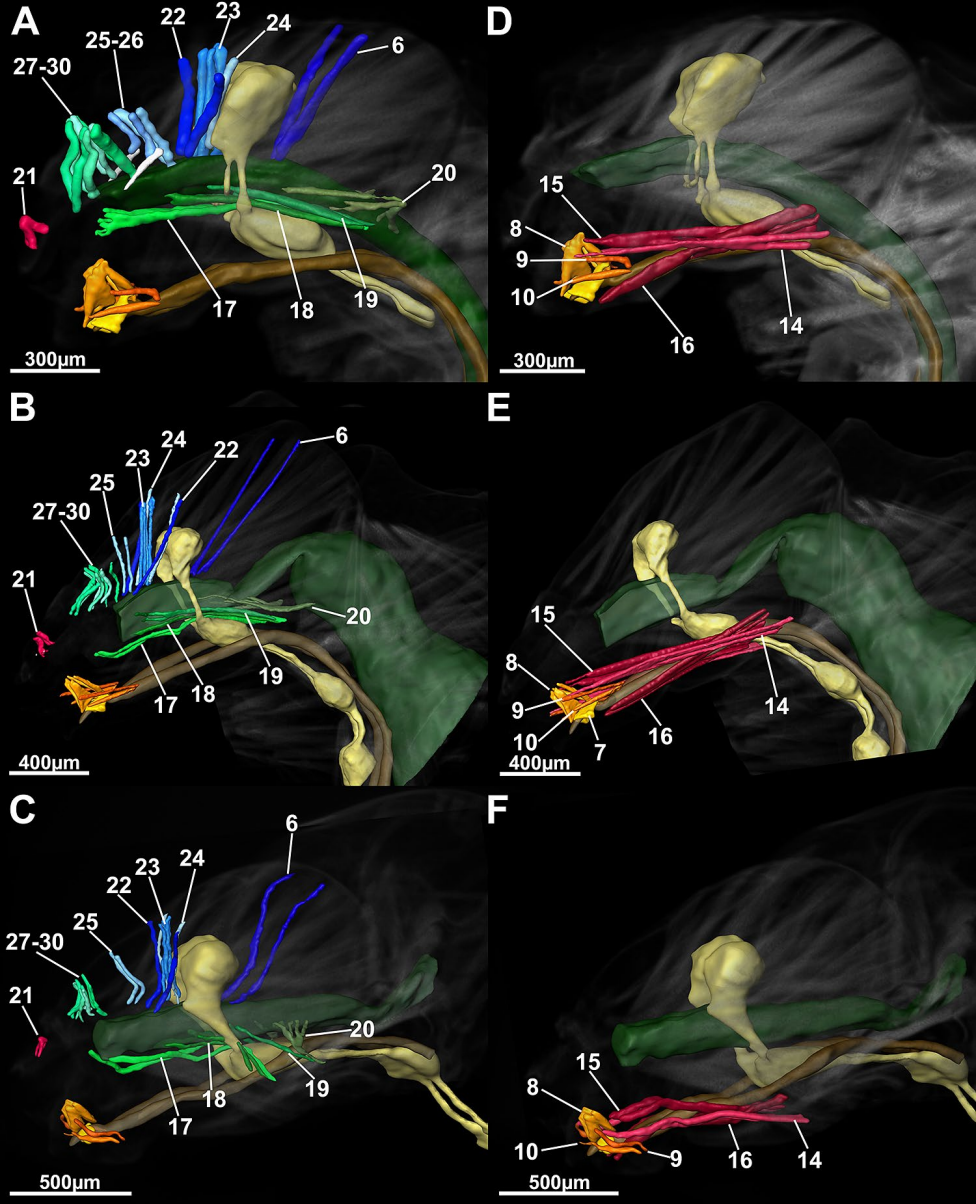
Lateral view of 5th instar larvae of *Drusus discolor* (**A**, **D**), *D. bosnicus* (**B**, **E**) and *D. franzi* (**C**, **F**) based on µCT data. Abbreviations: 6 = Musculus (M.) cranio-postpharyngalis dorsalis, 7 = M. praemento-salivaris, 8 = M. hypopharyngo-salivarialis, 9 = M. basistipito-dististipitalis lateralis, 10 = M. basistipito-dististipitalis medialis, 14 = M. cranio-dististipitalis, 15 = M. cranio-praementalis anterior, 16 = M. cranio-praementalis lateralis, 17 = M. cranio-cibarialis, 18 = M. cranio-pharyngalis anterior, 19 = M. cranio-pharyngalis posterior, 20 = M. cranio-postphyaryngalis ventralis, 21 = M. labro-epipharyngalis, 22 = M. cranio-pharyngalis lateralis, 23 = M. fronto-pharyngalis medialis, 24 = M. fronto-pharyngalis lateralis, 25 and 26 = M. fronto-pharyngalis ventralis, 27 and 28 = M. clypeo-pharyngalis, 29 and 30 = M. clypeo-cibarialis. The figure was built using the visualization software Amira 2020.2 (Thermo Fisher Scientific, https://www.thermofisher.com).

**Figure 7:**
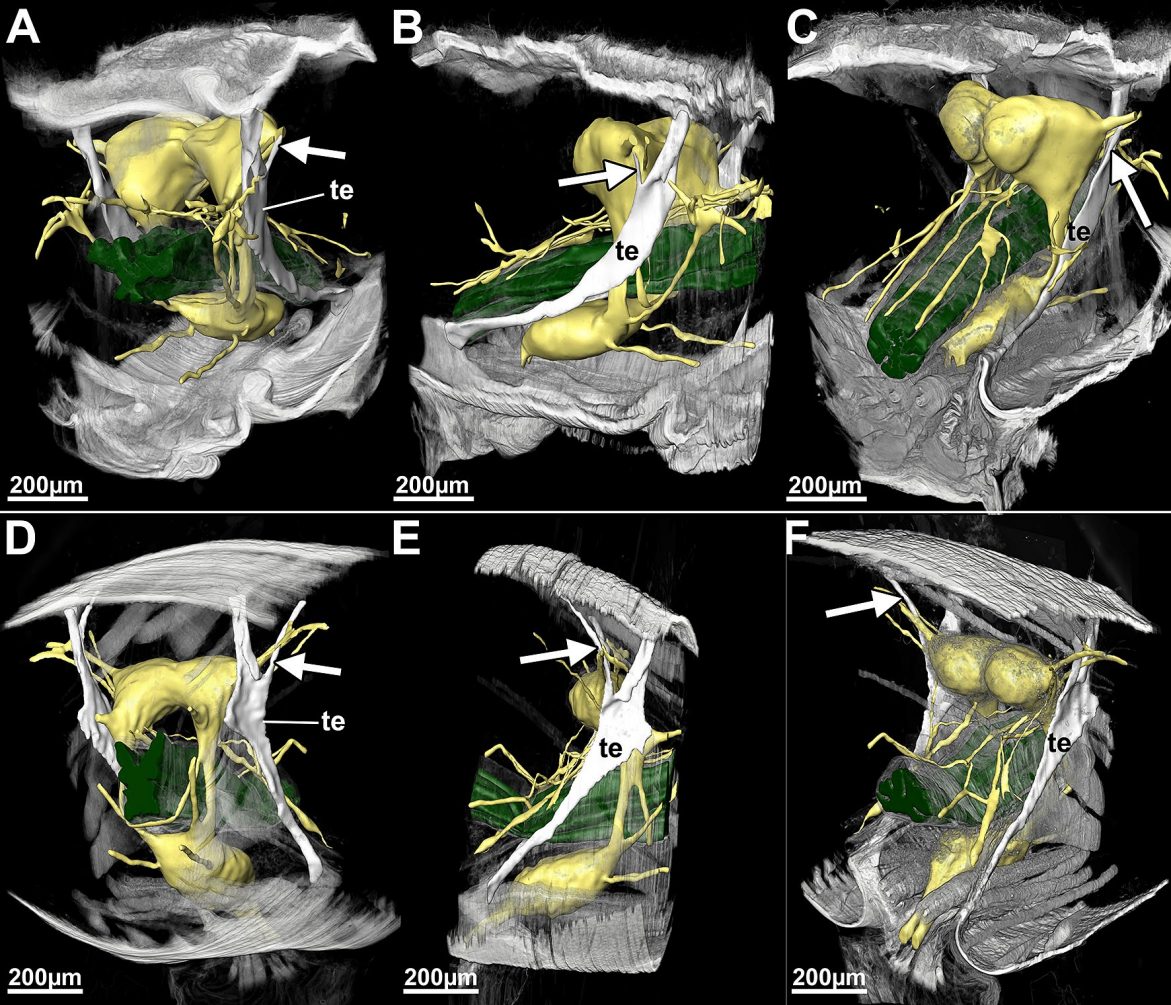
Visualization and comparison of the secondary supratentorial branch of *Drusus bosnicus* (**D**–**F**) which is missing in *D. discolor* (**A**–**C**) (indicated with an arrow) based on histological sections. Abbreviations: te = tentorium. central nervous system including cerebral ganglion mass, gnathal ganglion mass, frontal ganglion and innervation patterns in yellow. The figure was built using the visualization software Amira 2020.2 (Thermo Fisher Scientific, https://www.thermofisher.com).

Our second aim was to compare internal head anatomy among Drusinae clades. Despite the impressive differences in head capsule shape, each of the three species investigated here shares the same set of cephalic muscles, with only minute differences in the location of single points of origin of, e.g., frontal muscles (Figs. 5, 6). In particular, the points of origin relative to the M. fronto-labralis and the number of individual muscle bundles of the M. fronto-epipharyngalis differs among the species as well as the points of origin of the M. fronto-pharyngalis relative to the M. fronto-labralis. In *D*. *franzi*, the points of origin are arranged sequentially along the dorso-ventral plane in the following order: M. fronto-labralis, M. fronto-pharyngalis, M. fronto-epipharyngalis. In *D*. *discolor*, the same order of points of origin can be observed, where the M. fronto-pharyngalis is located somewhat closer to the M. fronto-pharyngalis, and the M. fronto-epipharyngalis has more than one point of origin on either side. *Drusus bosnicus* displays a different configuration where the dorsalmost points of origin of the M. fronto-labralis, the M. fronto-pharyngalis and the M. fronto-epipharyngalis are in roughly the same dorsoventral plane, the M. fronto-pharyngalis has more than one point of origin on either side, and the M. fronto-epipharyngalis has several points of origin that are located obliquely in sequence from the dorsalmost point of origin. Further, *D*. *discolor* exhibits a doubled M. fronto-pharyngalis and the points of origin of some muscle bundles of the M. cranio-mandibularis medialis differs among *D*. *discolor* and the two other investigated species. (Fig. 2, 3, 4). In contrast, all other muscles, including those of the alimentary canal and the maxillolabium, are highly similar in all three species (Figs. 5, 6). The only apparent internal change induced by the aberrant head morphology in *D*. *discolor* pertains to the tentoria, which lack a complete secondary supratentorial branch that is present in *D*. *franzi* and *D*. *bosnicus* (Figs. 5, 6, 7).

## Discussion

Head anatomy of the Drusinae appears to be highly conserved. The number and arrangement of head muscles are virtually identical in all investigated Drusinae species^18,19^. The duplication of a muscle pair in *D*. *discolor* (M. fronto-pharyngalis ventralis) is the only recognizable difference. Functionally, an engorging facultative predator such as the filtering carnivore *D*. *discolor* could benefit from greater mobility of the pharynx, but whether a single duplication or a somewhat larger volume of alimentary canal muscles can have that effect is doubtful.

Differences in muscle volumes however could reflect feeding ecology of Drusinae shredders and scraping grazers. The shredder *D. franzi*, consuming a tough, fibrous food source, may have relatively large mandible adductors that could enable stronger bites. Scraping grazers may be more limited in their food uptake, requiring a larger number of scraping movements per unit time so that larger mandible abductors in *D*. *bosnicus* may be an adaptation to this feeding mode by allowing for greater scraping frequency. While more comprehensive studies remain to be conducted, the preliminary data point towards the possibility that such volumetric differences can be observed: (1) Mandible adductors make up for roughly 85% of the total reconstructed head muscle volume in *D*. *franzi*, 77% in *D*. *discolor* and 72% in *D*. *bosnicus*; (2) the greatest mandible abductor muscle volume is in *D*. *bosnicus* (12% of the total head muscle volume); and (3) *D*. *discolor* has the greatest alimentary canal muscle volume (9% of total head muscle volume). These approximations may be a first indication for such a differentiation but remain to be verified in a larger, more standardized sample comprising several specimens of each species.

Drusinae head anatomy was first investigated in *D*. *trifidus*, a representative of the Drusinae grazer clade^18^. Data on another Drusinae grazer species, *D*. *monticola*, suggested high congruence of this species with the previously described condition^19^ but did not cover other evolutionary lineages of Drusinae. Here, we present evidence contrary to our initial hypotheses, suggesting largely identical head muscle number and arrangements in all three major evolutionary lineages of Drusinae with minor deviations in the filtering carnivore clade. Interestingly, the configuration of frontal muscles in the Drusinae grazer clade observed in *D*. *bosnicus* was also observed in the grazer *D*. *monticola*. While it is probable that the grazer *D. trifidus* exhibits the same pattern, the available data are insufficient for an assessment. Whether this configuration is typical for Drusinae scraping grazer remains to be evaluated, but this notion is conceivable because of the close relationships within this clade^13,17^.

Concerning the internal head skeleton, the tentorium, we posit that the changes of head shape in *D*. *discolor* and other filtering carnivorous Drusinae^13^ induce modifications such as an increasing simplification of the tentorium. In this regard, we assume that the modified head capsules of the filtering carnivore Drusinae^13,20^ offer greater mechanical stability due to their structured surface as compared to the rounded head capsules of the other Drusinae—thus, the dorsal branch of the tentorium is superfluous and can be reduced. We base this interpretation on the observed mechanical properties of corrugated bodies, that are capable of withstanding greater forces^21,22^. In-field measurements indicate that filtering carnivore larvae occupy microhabitats where hydraulic stress is higher compared to microhabitats of other Drusinae^23^. Adaptations increasing stability of particularly exposed body parts such as a corrugated head capsule may prove beneficial under such circumstances. However, head capsule shape in filtering carnivore Drusinae was previously interpreted in relation to flow modification around the larval head and feeding ecology. Flow patterns around Drusinae larval heads are the focus of ongoing research, but comparative analyses of mechanical properties of Drusinae head capsules remain to be conducted.

From a systematist point of view and pending further studies, the reduced dorsal branch of filtering carnivorous Drusinae tentoria could be a synapormorphy accompanying head capsule modification in this clade, evolving from a possibly plesiomorphic biramal tentorium of the Drusinae common ancestor.

The lack of differences in the internal anatomy of Drusinae heads that differ strongly in their outer head capsule morphology is surprising. We present the first data on a caddisfly larva with an aberrant head capsule shape, but whether our findings apply to other taxa remains to be investigated. A wide range of Trichoptera taxa develop larvae in which the head capsules are not in a simple round shape. Amongst the European Trichoptera, *Lithax niger* (Hagen 1859)is certainly one of the most distinctive forms, but to date no comparative morphological studies are present of this species. Likewise, there are no anatomical treatments on other species with aberrant head shapes. A suite of potential model taxa including Goeridae (*Silo* Curtis 1830, *Goera* Stephens 1829, *Lithax* Mclachlan 1876), Limnephilidae (filtering carnivorous *Drusus* Stephens 1837, *Philocasca* Ross 1941, *Pseudostenophylax* Martynov 1909), or Apataniidae (*Allomyia* Banks 1916) develop larval heads distinctly different from the rounded ones sported by their congeners. Whether the minor impact of head capsule modification on internal head anatomy can be confirmed in other taxa as well will be subject of future studies. The absence of major changes however suggests that head capsule modification is not a costly means of adaptation to specific habitats. Conversely, any change in cephalic musculature will inevitably affect feeding, gut movement or size and configuration of the central nervous system. Modified head capsules as observed in some Drusinae and other groups can therefore probably evolve quickly and at low evolutionary costs if less important areas of the cephalic exoskeleton are involved. Intriguingly, anecdotal evidence suggests that some other Trichoptera larvae with modified head capsules use high-stress hydraulic niches (e.g., *Allomyia*, pers. comm. J.J. Giersch).

Embryonic development of insect heads involves the formation of parietals and the frontoclypeus following a “bend and zipper” model^24^. Head appendage tissue (with the exception of the labrum) is not involved in this process, and the corresponding muscles are mesodermal derivatives that make contact with the epidermis during embryonic development^24,25^. Developmental gene expression regulates head capsule formation and shape, where gnathal appendages are formed under influence of pair-rule and Hox genes^24^. Processes and developmental genes controlling head capsule shape in Trichoptera are not known. Evidence from other insects with head capsule modifications, such as Scarabaeidae, suggest that sets of developmental factors are co-opted to act as controlling agents in horn formation^26,27^.

Assuming that the same or highly similar molecular controls of head capsule shape are used across the more homogeneous Trichoptera is therefore plausible. However, exact patterning and developmental mechanisms, and how development of species-specific head capsule shapes is maintained over time, remains obscure. In particular, comparative assessments within Drusinae as well as among different caddisfly families should be made to clarify the genomic background of head capsule corrugation and indentation. Most probably the same genes are involved in different families, but how exactly head capsule shapes take form during development and which ecological function head capsule shape and corrugation have is enigmatic. Available data on Drusinae hydraulic niches suggest that head capsule corrugation may be linked with high-stress microhabitats optimal for filter-feeding^23^. In other taxa (e.g., Goeridae, Brachycentridae, Apataniidae, etc.) head capsule corrugation and indentation may be the result of similar ecological constraints.

## Methods

### Sample preparation

Three Drusinae specimens of three different feeding and evolutionary clades, *Drusus bosnicus*, *D. discolor* and *D. franzi*, were used for µCT analysis. *Drusus discolor* was collected in the Schreierbach near Lunz am See, Ybbs catchment, Lower Austria (47˚50’ N; 15˚04’ E; 700 m. a. s. l.), on the 25th of July 1992 (leg. Johann Waringer). *Drusus bosnicus* was collected in the Paljanska Miljacka River, Bosnia-Herzegowina 2008, (43˚49′ N; 18˚32′ E; 848 m. a. s. l.) on 17th of May (leg. M. Kucinic).

*Drusus franzi* was collected at the Saualpe, Carinthia, Austria (46˚50′ N; 14˚40′ E; 1665 m. a. s. l.) on the 29th of May 2006 (leg. P. Wenzl). All samples were stored in 90% ethanol.

### µCT scanning

For µCT analysis, larvae were stained for 21 days in 1% (w/v) phosphotungstic acid (PTA) in 70% ethanol and washed in 70% ethanol to remove unbound PTA from tissue. Afterwards, the larvae were mounted vertically in 70% ethanol in the tip of a plastic pipette, and sealed with parafilm. Larvae were scanned on an XRadia MicroXCT-400 (Carl Zeiss X-ray Microscopy, Pleasanton, CA, USA) at 80kVp/ 100µA using the 4X detector assembly. Projections were recorded with 15 s exposure time (camera binning = 1) and an angular increment of 0.225° between projections over a 360° rotation. Tomographic slices were reconstructed with a voxel resolution of 2.87 µm (reconstruction binning = 1) using the XMReconstructer software provided with the µCT system.

### Image processing

The merged volume was exported as *.TXM file into Amira 2019.1 (FEI SAS, Mérignac, France (part of Thermo Fisher Scientific)). A 3D bilateral filter was used to filter the image volume for noise reduction. Image segmentation was achieved in Amira 6.5.0 (Visage Imaging, Inc., San Diego, CA, USA). Internal head anatomy (head muscles, tentoria, central nervous system including cerebral ganglion mass, gnathal ganglion mass, frontal ganglion and innervation patterns) were manually segmented and assigned to different “materials” within the segmentation editor. Three-dimensional surface renderings were created based on this manual segmentation using the Amira Surface Generate tool.

### Histology, computer-based 3D reconstruction and post processing

Heads of *D. bosnicus* and *D. discolor* were cut from the remaining body for histological processing. First samples were dehydrated with acidified dimethoxypropane followed by three rinses with acetone before being infiltrated and embedded in Agar LVR resin (Agar Scientific, Stansted, UK). Cure resin blocks were serially sectioned with a Diatome HistoJumbo diamond knife (Diatome, Nidau, Switzerland) at 1 µm section thickness on a Leica UC6 ultramicrotome (Leica microsystems, Wetzlar Germany). Sections were stained with 1% toluidine blue and sealed in epoxy resin. Analysis and photography of the serial sections was conducted on Nikon NiU compound microscope with a Nikon DsRi2 microscope camera (Nikon, Tokyo, Japan).

Image stacks were converted to greyscale and contrast-enhanced with F:IJI^28^ and subsequently imported into the visualization software (Thermo Fisher Scientific). Alignment of consecutive sections was conducted with the AlignSlices Tool of Amira. Structures of interest (tentorium, nervous system and digestive tract) were semi-manually reconstructed by labelling with a brush and interpolating several consecutive sections. Surfaces were calculated from the segmentation masks, followed by surface optimization using iterated smoothing and polygon-reduction steps. Snapshots were taken with the Amira software.

### Nomenclature and Drusus head capsule morphology

Nomenclature of cephalic muscles follows that for *D*. *trifidus*^18^ as updated by Friedrich and co-workers^28^, with modifications to reflect conditions in Drusinae that differ from those in *Rhyacophila fasciata* Hagen 1859. This pertains to (i) the M. tentorio-cibarialis and the M. tentorio-pharyngalis anterior and posterior sensu Friedrich and co-workers—for which we use the terms M. cranio-cibarialis and M. cranio-pharyngalis anterior and posterior as the origin of these muscles is not on the tentorium but rather close to its base on the parietals; (ii) the M. cranio-pharyngalis dorsalis and ventralis sensu Friedrich and co-workers—here, we use the terms M. cranio-postpharyngalis dorsalis and ventralis to describe their location somewhat more precisely.

Head capsule morphology of *D*. *trifidus* is described elsewhere^18^. Other than head capsule shape, the following differences to the condition found in *D*. *trifidus* were observed: in *D*. *franzi* and *D*. *discolor*, the mandibles bear three terminal teeth and the antenna lacks a distinct base^13,14,20,29^ whereas in D. *bosnicus* the head capsule is flattened at the vertex and the antennal carinae are longer than in *D*. *trifidus*^14,30^. Comparative studies on homologous structures of larval head anatomy and morphology across Trichoptera and other groups are outside the scope of this work, but are available elsewhere^31,32^.

## Data Availability

All data generated or analyzed during this study are included in this published article.
